# Effects of financial support on treatment of adolescents with growth hormone deficiency: a retrospective study in Japan

**DOI:** 10.1186/s12913-016-1854-z

**Published:** 2016-10-21

**Authors:** Eri Maeda, Takahiro Higashi, Tomonobu Hasegawa, Susumu Yokoya, Takahiro Mochizuki, Tomohiro Ishii, Junko Ito, Susumu Kanzaki, Akira Shimatsu, Koji Takano, Toshihiro Tajima, Hiroyuki Tanaka, Yusuke Tanahashi, Akira Teramoto, Toshiro Nagai, Kunihiko Hanew, Reiko Horikawa, Toru Yorifuji, Naohiro Wada, Toshiaki Tanaka

**Affiliations:** 1 Department of Environmental Health Sciences, Akita University Graduate School of Medicine, 1-1-1 Hondo, Akita-shi, Akita 010-8543 Japan; 2 Division of Health Services Research, Center for Cancer Control and Information Services, The National Cancer Center, 5-1-1 Tsukiji, Chuo-ku, Tokyo, 104-0045 Japan; 3 GH Treatment Study Committee, The Foundation for Growth Science, 5-1-16 Hongo, Bunkyo-ku, Tokyo, 113-0033 Japan

**Keywords:** Public funding, Health insurance, Children with special health care needs, Japan

## Abstract

**Background:**

Treatment costs for children with growth hormone (GH) deficiency are subsidized by the government in Japan if the children meet clinical criteria, including height limits (boys: 156.4 cm; girls: 145.4 cm). However, several funding programs, such as a subsidy provided by local governments, can be used by those who exceed the height limits. In this study, we explored the impacts of financial support on GH treatment using this natural allocation.

**Methods:**

A retrospective analysis of 696 adolescent patients (451 boys and 245 girls) who reached the height limits was conducted. Associations between financial support and continuing treatment were assessed using multiple logistic regression analyses adjusting for age, sex, height, growth velocity, bone age, and adverse effects.

**Results:**

Of the 696 children in the analysis, 108 (15.5 %) were still eligible for financial support. The proportion of children who continued GH treatment was higher among those who were eligible for support than among those who were not (75.9 % vs. 52.0 %, *P* < 0.001). The odds ratios of financial support to continuing treatment were 4.04 (95 % confidence interval [CI]: 1.86–8.78) in boys and 1.72 (95 % CI: 0.80–3.70) in girls, after adjusting for demographic characteristics and clinical factors.

**Conclusions:**

Financial support affected decisions on treatment continuation for children with GH deficiency. Geographic variations in eligibility for financial support pose an ethical problem that needs policy attention. An appropriate balance between public spending on continuation of therapy and improved quality of life derived from it should be explored.

## Background

Biosynthetic human growth hormone (GH) is very expensive. The annual cost for one child weighing 30 kg is reported to be approximately US $15,000 to $24,000 [1–3]. Although most of the costs are covered by public health insurance or reimbursed by governments in many countries [4], the extent to which public funding should be used for support involves ethical issues, resulting in different policies [1, 4]. Little is known about the impact of financial support on decision-making for treatment of children with growth hormone deficiency (GHD).

**Fig. 1:**
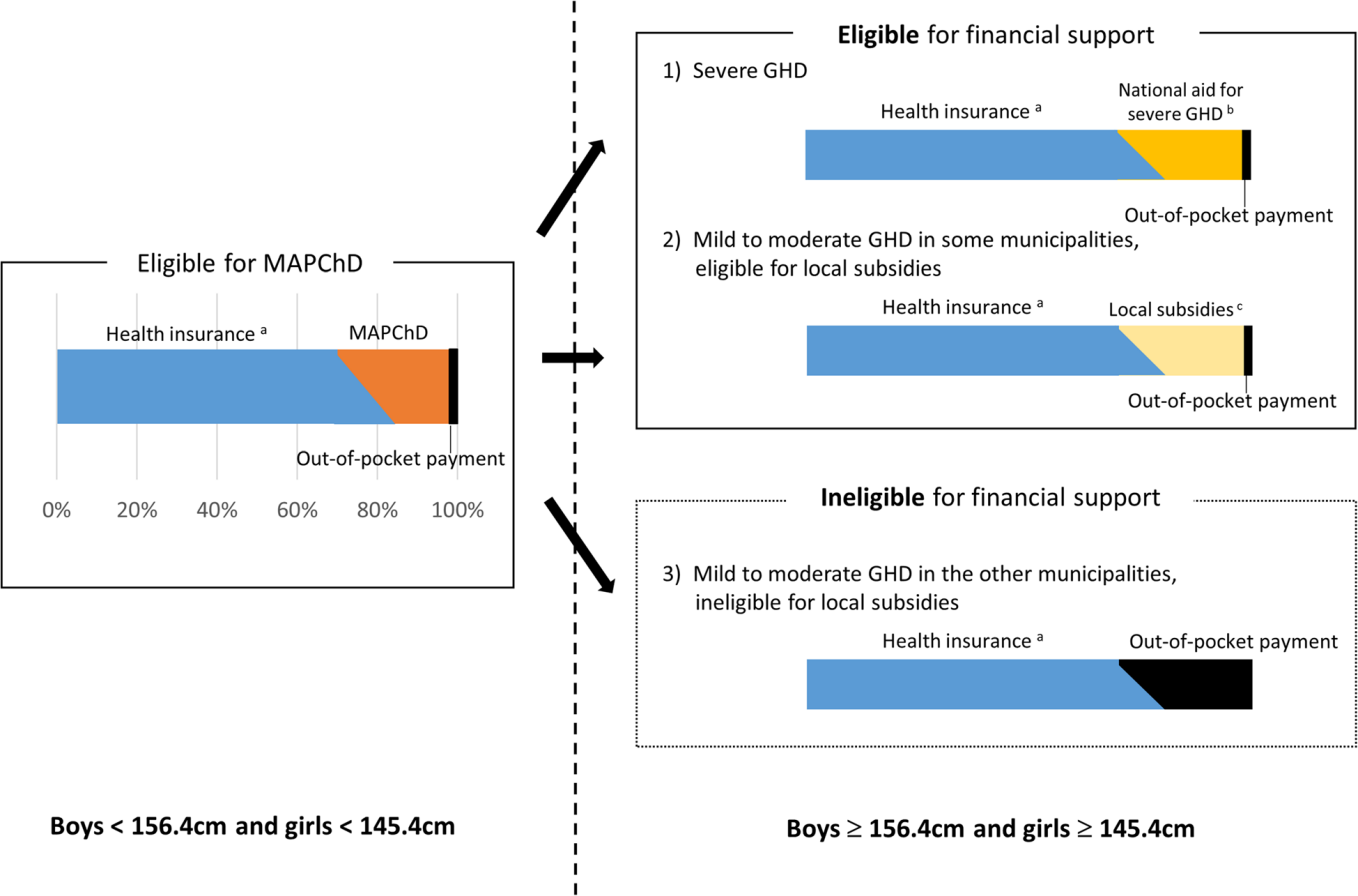
The proportion of the medical charges paid by insurance, subsidies, and patients’ out-of-pocket payments. GHD, growth hormone deficiency; MAPChD, Medical Aid Program for Chronic Pediatric Diseases of Specified Categories. ^a^Health insurance covers 70 % of the medical charges at baseline and provides a monthly cap on family out-of-pocket payment, depending on the annual household income. Because many GHD patients reach the cap, the combination is expected to result in total coverage of up to about 85 % of total medical expenses among GHD children, as represented by the oblique line of coverage levels. ^b^The national aid for severe GHD was established in October 2009. ^c^Local subsidies include a local subsidy for mild GHD and the generic Child Health Insurance Subsidy program

Important lessons may be learned by studying the case of Japan, where the level of subsidy for treatment depends purely on the patient’s geographic location (Fig. 1). Generally, Japan provides universal health insurance coverage to its citizens up to 70 % of the medical charges, with the remainder paid by patients [5]. However, for some rare diseases, including GHD, national and local subsidies provide financial support for patients’ out-of-pocket payments. Doctors ensure that patients understand what programs are available.

The primary program to assist children with GHD is the Medical Aid Program for Chronic Pediatric Diseases of Specified Categories (MAPChD) [6, 7]. This program covers most of the copayments for children with GHD who meet certain criteria. These criteria include height limits up to a −2.5 standard deviation score (SDS) of the height for near-adults (i.e., 17.5-year-old children); specifically, 156.4 cm for boys and 145.4 cm for girls. Another program, established by the national government in October 2009, is the aid for severe GHD, which covers treatment costs for patients who exceed the eligible height limits for MAPChD [8]. Local governments operate two other programs. One is a subsidy offered by some municipalities for children with mild GHD who do not meet either of the subsidy criteria defined by the national government. The other is the generic Child Health Insurance Subsidy program, which is available in all 1742 municipalities in Japan. This program covers medical copayments of children who live in these municipalities, regardless of their health. While the MAPChD uniformly covers every patient in Japan, the eligibility criteria and benefits of local programs vary across municipalities [9]. Some municipalities cover children up to only 3 years of age, whereas others offer coverage up to 22 years of age. Copayments can range from free to a few dollars per visit, depending on household income. Therefore, some children with GHD who exceed the MAPChD height limit can receive financial support, but others cannot, depending on where they live.

The present study used this geographic variation in subsidy access as a natural experiment and examined the impacts of financial support on GH treatment. Based on earlier works regarding costs and treatment-seeking behavior [10–18], we hypothesized that children with GHD who were eligible for financial support from local programs were more likely than those who were ineligible for any financial support to continue GH treatment even after they reached the height limit and were no longer subsidized by the MAPChD. Insurance coverage generally has positive effects on the use of pediatric specialty care in the United States [10–16], but health economic literature on pediatric care is very limited in Japan [17, 18]. The aims of the study were to evaluate the relationship between financial support and patient behavior among children with GHD and to reveal possible effects of the geographical variation of local subsidy programs on access to specialty care among children in Japan.

## Methods

### Study population

The study population consisted of children with GHD registered in the Foundation for Growth Science database [19]. The Foundation for Growth Science works closely with the government to regulate GH use in Japan. Until 1997, registration with the foundation was required to apply for MAPChD, but this requirement has been discontinued. Currently, clinicians across the nation voluntarily register patient data in the database, and the registration rate of children with GHD is estimated to be 30 % [19]. The registry database includes patient age, sex, clinical data (e.g., height, weight, bone age, puberty status, results of GH tests, adverse effects), treatment protocols, whether patients continued or stopped GH, and the locations of medical institutions.

Registry data included 2471 children who reached the MAPChD height limits (for boys, 156.4 cm; for girls, 145.4 cm) from January 2001 to May 2013. After excluding children who did not meet the clinical criteria for MAPChD eligibility in the first place, we analyzed data on 696 children (451 boys and 245 girls) who met the criteria at the first registration and then became ineligible because they reached the height limits. Criteria for MAPChD are as follows. The criteria for starting GH in cases without organic abnormalities are height ≤ −2.5 SDS; insulin-like growth factor1 (IGF-1) < 200 ng/ml or < 150 ng/ml in children < 5 years old; and peak GH in provocative test ≤ 6 ng/ml. The criteria for starting GH in cases with organic abnormalities are height ≤ −2.0 SDS or growth velocity (GV) ≤ −1.5 SDS for 2 years; and peak GH in provocative test ≤ 3 ng/ml. The criteria for continuing GH in the first treatment year are GV ≥ 6.0 cm/year or GV increment ≥ 2.0 cm/year. The criterion for continuing GH in the second year and after is GV ≥ 3.0 cm/year.

###  Financial support for patients taller than the MAPChD height limits

We categorized children who met the criteria for severe GHD and reached the MAPChD height limit after October 2009, when the aid for severe GHD was established, as those who were eligible for the national subsidy for severe GHD. Criteria for severe GHD included a peak GH in provocative tests of ≤3 ng/ml in children with height ≤ −2.0 SDS or a peak GH ≤ 1.8 ng/ml in adults.

We collected information on the local subsidy programs for the children in the sample, including the presence or absence of programs. If a program existed, we recorded the eligibility criteria either by searching program web sites or by contacting the Ministry of Health, Labour and Welfare, prefectures, and municipalities by telephone or e-mail. Collating the age of each patient and the eligibility criteria for the local subsidies in each municipality, we categorized children into two groups (eligible and ineligible), depending on whether they were eligible for at least one of the national or local subsidies.

### Data analyses

Statistical comparisons were carried out to compare the variables between patients who were eligible or ineligible for financial support, using Student’s *t* tests, two-group variance-comparison tests, Welch’s *t* tests, Wilcoxon’s rank sum tests, chi-square tests, and Fisher’s exact tests, according to the type of outcome variables and distribution of the variables. Multiple logistic regression analysis was used to assess the relation of financial support to the continuation of GH treatment, after adjusting for possible confounders: age in years, sex, height (on a standard deviation scale; SDS), growth velocity (centimeters per year), bone age in years, adverse effects (yes/no), and when patients reached height limits (before or after October 2009, when the national subsidy for severe GHD was implemented). We assessed interaction terms between sex and financial support and between sex and bone age to account for possible differences in the effects of financial support and bone age on the continuation of therapy between boys and girls.

We also conducted sensitivity analyses to test the robustness of our findings. First, we removed bone age from the regression model, because 76 values were missing for bone age and because age and bone age were moderately correlated (Pearson’s correlation coefficient: 0.53, *p* < 0.001). Second, we included pubertal status (pre- or post-puberty) in the regression model. Finally, to address the possibility that treatment decisions were changed after the data were registered, we excluded children from the treated group if they were not registered as treated patients in the following year. All analyses were performed using Stata12-SE (StataCorp LP, College Station, TX, USA).

## Results

Of the 696 children, 42 (6.0 %) were eligible for the national subsidy for severe GHD. Regarding local subsidies, medical institutions of the 696 children were distributed across 155 municipalities (8.9 % of the total 1742 municipalities in Japan). Of these children, 69 children in 22 municipalities and three children in two municipalities were assumed to meet criteria for the local Child Health Insurance Subsidies and the local subsidy for mild GHD, respectively. There were six children who were eligible for both of the national and local subsidies. Overall, 108 children (15.5 %) were categorized as eligible for financial support after they reached the MAPChD height limits.

**Fig. 2:**
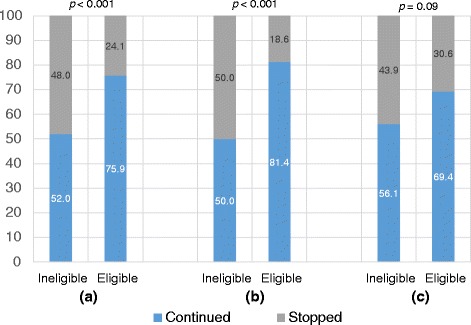
The proportion of patients who continued treatment, by financial support status and by sex: **a** present sample; **b** boys; **c** girls

Table 1 lists the characteristics of the children. The proportion of children who continued GH was higher among those with financial support than among those without (75.9 % vs. 52.0 %, *p* < 0.001, Fig. 2). The percentage of children who were eligible for financial support was significantly higher among girls.

**Table 1:** Characteristics of the 696 patients and additional financial support after reaching the height limit

Variables	Ineligible	Eligible^a^	*p* value^b^
	(*n* = 588)	(*n* = 108)	
GH treatment, *n* (%)			
Stopped	282 (48.0)	26 (24.1)	<0.001
Continued	306 (52.0)	82 (75.9)	
Sex, *n* (%)			
Girls	196 (33.3)	49 (45.4)	0.016
Boys	392 (66.7)	59 (54.6)	
Age, y			
Mean (SD)	14.8 (1.4)	14.4 (1.3)	0.0015
Girls	13.7 (1.0)	13.7 (1.0)	0.62
Boys	15.4 (1.2)	15.0 (1.1)	0.017
Height SDS			
Median	−1.59	−1.525	0.16
(Interquartile range)	(−1.89 to −1.21)	(−1.81 to −1.07)	
Growth velocity, cm/y			
Mean (SD)	6.7 (2.9)	6.5 (2.5)	0.46
Bone age, y	(*n* = 520)	(*n* = 100)	
Mean (SD)	13.1 (1.4)	12.9 (1.2)	0.13
Girls	12.1 (1.1)	12.2 (1.0)	0.82
Boys	13.6 (1.3)	13.6 (0.9)	0.56
Puberty status, *n* (%)	(*n* = 562)	(*n* = 108)	
Pre-puberty	68 (12.1)	17 (15.7)	0.30
Post-puberty	494 (87.9)	91 (84.3)	
Adverse effects, *n* (%)			
No	560 (95.2)	105 (97.2)	0.46
Yes	28 (4.8)	3 (2.8)	
When patients reached height limit, *n* (%)			
Before October 2009	437 (74.3)	9 (8.3)	<0.001
October 2009 and after	151 (25.7)	99 (91.7)	

*GH* growth hormone, *SD* standard deviation, *SDS* standard deviation score

^a^Eligible for at least one of the national or local programs of financial support after the child exceeded the height limit defined by the Medical Aid Program for Chronic Pediatric Disease of Specified Categories

^b^Chi-square tests, two-tailed *t*-tests, Wilcoxon rank sum tests, Welch *t*-tests, or Fisher’s exact tests

As shown in Table 2, multiple logistic regression analysis revealed that eligibility of financial support was associated with 4.04 (95 % confidence interval [CI]: 1.86–8.78) times the odds of continuing treatment among boys, and the corresponding odds among girls were as small as 1.72 (95 % CI: 0.80–3.70). The percentages of those who continued GH treatment were 76.7 % among those with financial support and 54.7 % among those without, after adjusting for the previously mentioned factors (80.9 % vs. 52.9 % among boys and 69.4 % vs. 58.0 % among girls).

**Table 2:** Multiple logistic regression analysis for factors related to treatment decisions (*n* = 620)

Variables	OR	95 % CI
Eligibility for financial support^a^		
Ineligible	Reference	
Eligible (girls)	1.72	0.80–3.70
Eligible (boys)	4.04	1.86–8.78
Sex^b^		
Girls	Reference	
Boys	1.88	1.00–3.51
Age, y		
Each additional year older	0.64	0.51–0.79
Height SDS		
Each additional SDS increase	0.65	0.43–0.99
Growth velocity, cm/y		
Each growth velocity increase	1.04	0.97–1.12
Bone age, y^c^		
Each additional year older (girls)	0.69	0.52–0.93
Each additional year older (boys)	0.90	0.74–1.09
Adverse effects		
No	Reference	
Yes	0.61	0.28–1.32
When patients reached the height limit		
Before October 2009	Reference	
October 2009 and after	0.84	0.56–1.26

*CI* confidence interval, *OR* odds ratio

^a^Eligibility for at least one of the national or local programs of financial support after exceeding the height limit defined by the Medical Aid Program for Chronic Pediatric Disease of Specified Categories

^b^Comparing boys at the bone age of 15 y to girls at the bone age of 13 y, without financial support (comparing different bone ages due to differential centering for the variable)

^c^Bone age was centered at 13 y in girls and at 15 y in boys

Table 3 shows the results of the sensitivity analyses when we removed bone age from the baseline model, when we added pubertal status to the model, and when we excluded the cases in which treatment decisions might have changed after the data were registered. The ORs of the first and second analyses are similar to those in the original model. In the third analysis, 136 patients were registered to continue GH but were not registered as being treated in the following year. When we excluded them as unknown cases, the ORs were 2.91 and 2.37 in boys and girls, respectively.

**Table 3:** Sensitivity analyses of the odds ratio of financial support to continuing treatment

	Boys		Girls	
	OR	95 % CI	OR	95 % CI
Base model (*n* = 620)	4.04	1.86–8.78	1.72	0.80–3.70
Excluding bone age (*n* = 696)	4.31	2.05–9.03	1.99	0.95–4.17
Including puberty status (*n* = 602)	4.11	1.88–8.96	1.76	0.81–3.80
Excluding patients not registered in the following year (*n* = 491)	2.91	1.21–6.97	2.37	0.99–5.67

*CI* confidence interval, *OR* odds ratio

## Discussion

Our findings revealed that 76.7 % of children with GHD who were financially supported continued treatment even after they exceeded the government-defined height criteria, whereas only 54.7 % of those who were not supported continued GH. Although previous studies have shown that financial support generally promotes treatment seeking in patients [10–18], this study is the first to assess the effects of financial support on GH treatment. Importantly, the percentage of children with GHD who reached the height limit and stopped treatment for reasons other than financial factors was less than 25 %. Given the strong impact of public funding on treatment decisions, the clinical criteria for MAPChD eligibility of GHD patients must be carefully considered and based on concrete evidence. Although direct generalization to other countries may be difficult because of the different health care systems, our findings can support policy discussion not only in Japan but also in other countries that financially support GH treatment [4].

In the present sample, financial support had larger effects on boys than on girls. Differences in clinical characteristics (i.e., hormonal levels [20] or treatment response [21]) might be possible, although we made adjustment for the available clinical factors. Another possibility is the gender difference in attitudes toward height. Our data might have reflected patients’ behavior based on societal preferences for tall males, as shown in earlier works [22–24]. However, a more likely reason is the age criteria for the local Child Health Insurance Subsidies. Most municipalities provide a subsidy up to 15 years of age, at which point girls typically experience a natural slowing of growth in height, whereas boys are still rapidly growing [25]. This fact may have influenced the girls’ decisions to discontinue therapy. Although we cannot test this hypothesis because of the limited number of sample patients in respective municipalities that had different criteria, consideration of the sex differences in growth may be needed when setting the criteria for future subsidies.

Our results are consistent with those of prior studies describing health care improvement among children with special health care needs [26] after enrollment in the State Children’s Health Insurance Program in the United States [10–15]. Both in specific populations, such as patients with asthma [12, 13], and in heterogeneous populations with a wide range of health statuses, such as children with special health care needs [11, 14, 15], a decline in unmet health care needs and an increase in utilization were observed. A study using data from the RAND Health Insurance Experiment [16] showed a 36 % decline in outpatient episodes for chronic care among children assigned to cost-sharing plans, compared with those on a free care plan.

The present findings also agree with previous research in Japan, although the few available studies were restricted to general care. These studies showed that local subsidy programs directed parental attitudes toward immediate visit [17] and increased the probability of receiving treatment, especially among children aged between 7 and 12 years [18]. With regard to specialty care for children with rare diseases, the current study was the first to describe the association between local subsidy programs and care-seeking behavior. Although little attention has been paid to geographical differences in financial support in Japan, we found that 22 % more patients stopped treatment because of a lack of financial support compared with patients that had financial support. This geography-based inequality raises an ethical concern. Currently, MAPChD [6, 7] supports out-of-pocket payment of children with 704 chronic diseases [27] and defines subsidy criteria for some of the diseases, such as GHD. In view of the previous results that parental attitudes toward severe symptoms of colds were not significantly different regardless of the level of subsidies [17], caution should be taken in applying the present results to other disease that have different clinical features and payment amounts. However, this study provides policy-makers with an important reference to resolve this geographic gap.

Establishing evidence-based criteria for public funding in GH treatment is an area for future research. The current height limit does not seem to meet patients’ needs. The mean height velocity of the present population was 6.7 cm/year, and the mean bone ages were 13.6 and 12.1 years old in boys and girls, respectively. According to the longitudinal data of Japanese school children [25], most children in the present sample were at peak height velocity and could grow closer to normal heights. However, a previous cohort study showed that patients who stopped treatment early spontaneously gained 0.4 SDS after the end of treatment, possibly because of the difficulty in distinguishing GHD from pubertal delay [28]. Determining endpoints of treatment is difficult in the majority of patients without severe GHD [1]. Treatment appears to be effective [2, 29, 30] and safe [31] in many cases, but careful follow-up surveys for adult height would provide more information about the extent to which the public should support patients with GHD.

Furthermore, constructing cost-effectiveness models based on pertinent evidence regarding adult height, other health outcomes, and quality of life may help in confirming or revising current criteria although so far, evidence based on long-term follow-up is limited [28, 32]. Previous work has calculated the incremental cost effectiveness of GH treatment at approximately £6000 (about US $9000, using a 2002 exchange rate) per centimeter gained in adult height [33]. In addition to height, we should also include metabolic effects [34, 35], psychological effects [36–39], and short-term [40] and long-term quality of life [41–44], all of which remain inconclusive and need further research.

This study has several limitations. First, the use of a nonmandatory registry database could have caused a selection bias. Although cases of discontinuation might be less likely to be registered than those of continuation, there is no reason to consider the registry to be affected by the recipient status of financial support. Second, the registry did not contain socio-economic information about the patients. Future surveys should address the influence of these factors for in-depth understanding of the process of patients’ decision-making. Third, some patients may have been misclassified as eligible for local subsidies. We determined the presence or absence of local subsidies based on the locations of the treatment providers, because the data set did not contain patients’ residences. Also, we did not account for differences in benefits across municipalities, income-based eligibility limit, or the presence of other subsidies, such as subsidies for single parents or for the disabled [45]. However, these factors would not have largely influenced our results. Any misclassification caused by substituting patients’ residences with locations of medical institutions would have had a negligible effect on the results because the eligibility criteria defined by neighboring municipalities are usually similar under financial help by prefectures [9]. The difference in benefits is relatively small compared with the high cost of GH. Furthermore, misclassification of financial support eligibility criteria in this study would likely result in the underestimation of the relationship in two ways. Income-based eligibility limit would prevent patients who otherwise satisfy criteria from receiving support, making some patients who appeared to be “eligible” for support more likely to have discontinued therapy. Similarly, the presence of other subsidies not considered in this study may have increased the likelihood of patients continuing therapy despite being categorized as “ineligible”.

## Conclusions

This study revealed that financial support affects decision-making for treatment of adolescents with GHD. Because cost is a major consideration in GH treatment, future cost-effectiveness analyses addressing quality-of-life changes as well as physical and psychological improvement are needed to establish more evidence-based criteria for public funding.

## Abbreviations

GH: Growth hormone
GHD: Growth hormone deficiency
MAPChD: Medical Aid Program for Chronic Pediatric Diseases of Specified Categories
OR: Odds ratio
SDS: Standard deviation score
